# Simulation modeling of breast cancer endocrine therapy duration by patient and tumor characteristics

**DOI:** 10.1002/cam4.4084

**Published:** 2021-12-16

**Authors:** Young Chandler, Clyde Schechter, Jinani Jayasekera, Claudine Isaacs, Allison W. Kurian, Christopher Cadham, Jeanne Mandelblatt

**Affiliations:** ^1^ Department of Oncology Georgetown‐Lombardi Comprehensive Cancer Center Washington DC USA; ^2^ Albert Einstein College of Medicine Montefiore Medical Center Bronx NY USA; ^3^ Department of Medicine Georgetown‐Lombardi Comprehensive Cancer Center Washington DC USA; ^4^ Departments of Medicine and Epidemiology and Population Health Stanford University Stanford CA USA

**Keywords:** adjuvant therapy, adverse events, breast cancer, endocrine therapy, simulation modeling

## Abstract

**Background:**

Extending endocrine therapy from 5 to 10 years is recommended for women with invasive estrogen receptor (ER)‐positive breast cancers. We evaluated the benefits and harms of the five additional years of therapy.

**Methods:**

An established Cancer Intervention and Surveillance Network (CISNET) model used a lifetime horizon with national and clinical trial data on treatment efficacy and adverse events and other‐cause mortality among multiple birth cohorts of U.S. women ages 25–79 newly diagnosed with ER+, non‐metastatic breast cancer. We assumed 100% use of therapy. Outcomes included life years (LYs), quality‐adjusted life years (QALYs), and breast cancer mortality. Results were discounted at 3%. Sensitivity analyses tested a 15‐year time horizon and alternative assumptions.

**Results:**

Extending tamoxifen therapy duration among women ages 25–49 reduced the lifetime probability of breast cancer death from 11.9% to 9.3% (absolute difference 2.6%). This translates to a gain of 0.77 LYs (281 days)/woman (undiscounted). Adverse events reduce this gain to 0.44 QALYs and after discounting, gains are 0.20 QALYs (73 days)/woman. Extended aromatase inhibitor therapy in women 50–79 had small absolute benefits and gains were offset by adverse events (loss of 0.06 discounted QALYs). There were greater gains with extended endocrine therapy for women with node‐positive versus negative cancers, but only women ages 25–49 and 50–59 had a net QALY gain. All gains were reduced with less than 100% treatment completion.

**Conclusion:**

The extension of endocrine therapy from 5 to 10 years modestly improved lifetime breast cancer outcomes, but in some women, treatment‐related adverse events may outweigh benefits.

## Graphical abstract

Simulation modeling was used to evaluate extended endocrine therapy for women with hormone receptor‐positive non‐metastatic breast cancer. Results indicate that extension of endocrine therapy from 5 to 10 years modestly improves life years in some groups but adverse events may outweigh benefits.

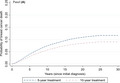

## 1 INTRODUCTION

The majority of U.S. women diagnosed with breast cancer have estrogen receptor‐positive (ER+) tumors.^1^ These women have high survival rates, but remain at risk for distant recurrences. Extending endocrine treatment from 5 to 10 years can decrease recurrence and second contralateral breast cancers^2^, ^3^, ^4^ and is now recommended as standard care.^5^ Clinical guidelines also stress the need to personalize treatment duration since absolute gains can vary by agents, are small^2^ and might not be realized in community settings,^6^, ^7^, ^8^ and could be offset by side effects.^9^, ^10^, ^11^

However, it can be difficult to estimate the net impact of benefits and adverse events for individual women considering their age, screening history, and tumor characteristics. We used an established Cancer Intervention and Surveillance Modeling Network (CISNET) simulation model^12^, ^13^ to combine data on individual and tumor characteristics to determine lifetime outcomes by the duration of endocrine therapies among groups of U.S. women. The results are intended to illustrate how modeling can support personalized shared decision‐making discussions between patients and oncologists about the net balance of extended endocrine therapy.

## 2 METHODS

The Georgetown University Institutional Review Board approved this modeling research as exempt based on the use of de‐identified, publicly available data.

### 2.1 Model overview

CISNET Model G‐E is one of the six breast cancer models and has previously been described.^12^, ^13^ Model G‐E was selected for this study because it is a representative CISNET model, the structure is well suited to evaluating alternative treatment options and it replicates SEER trends. Briefly, Model G‐E is a continuous‐time event‐driven micro‐simulation. For this study, the model included cohorts of U.S. women born from 1935 to 2009 ages 25–79 when diagnosed with ER+stage 1–3 breast cancer. The model started with basic life history for each woman in the absence of adjuvant therapy, assuming population screening patterns. Life histories were then repeated with endocrine therapy (5 or 10 years) and other indicated systemic therapies, with treatment modifying time to and probability of breast cancer death.^13^

### 2.2 Input parameters

We used national and clinical trial data to develop model input parameters (Table 1). Age‐period‐cohort analyses were used to estimate breast cancer incidence rates in the absence of screening.^14^ National survey data were used to estimate screening use by birth cohort. Digital mammography performance characteristics were derived by age group (<50 vs. 50+), first versus subsequent screening, and time since last mammogram using Breast Cancer Surveillance Consortium (BCSC) data as previously described.^15^ These data were used to generate women diagnosed with incident breast cancer. All women receive local therapy with surgery with or without radiation therapy.

**TABLE 1:** Model input parameters

Parameter	Value/Range/Description					Source	
Births	Cohorts born 1935–2009, age 25–79					[34]	
Incidence	Age‐period‐cohort model					[14]	
Mammography use	Screening patterns by birth cohort						
Mammography sensitivity	Age‐specific rates for first and subsequent screens					[12]	
Subtype distribution	The probability of each subtype conditional on age, stage, and screening					[12]	
Age‐specific general health utilities							
20–29 years	0.913					[24]	
30–39 years	0.893					[24]	
40–49 years	0.863					[24]	
50–59 years	0.837					[24]	
60–69 years	0.811					[24, 35]	
70–79 years	0.771					[24]	
80+ years	0.724					[24]	
Stage‐specific utility							
Stage 1	0.9					[15, 36]	
Stage 2a	0.85						
Stage 2b	0.85						
Stage 3	0.8						
Stage 4	0.4						
Treatment effectiveness (HR‐ reduction in breast cancer mortality), conditional on stage, subtype							
	5 years		10 years				
Age 25–49, stage 1							
Tamoxifen	0.70 (0.66–0.88)		0.54 (0.51–0.68)			[2, 17]	
Age 25–49, stages 2/3							
Tamoxifen (with ovarian suppression if node +)	0.66 (0.62–0.84)		0.50 (0.47–0.64)			[16, 37]	
Age 50–79, stages 1–3							
Aromatase inhibitor	0.55 (0.46 −0.72)		0.54 (NE)^1^			[16, 18]	
Probability of grade 3–4 adverse events by regimen and treatment duration^2^, ^3^							
Treatment duration	5 years (0 to 5)		10 years (0 to 10)				
Regimen	tamoxifen	tamoxifen +ovarian suppression	aromatase inhibitor	tamoxifen	tamoxifen +ovarian suppression	aromatase inhibitor	
Adverse event							
Stroke	1.85% (1.75%–2.17%)	1.85% (1.68%–2.02%)	1.85% (1.69%–1.98%)	2.01% (1.67%–2.48%)	2.01% (1.84%–2.21%)	2.01% (1.85%–2.13%)	[2, 38]
Pulmonary embolus	3.03% (2.86%–3.56%)	2.3% (2.09%–2.51%)	0.83% (0.76%–0.89%)	5.87% (4.89%–7.26%)	4.46 (4.08%–4.90%)	0.8% (0.74%–0.85%)	[2, 21, 38, 39]
New cardiac condition	2.53% (2.39%–2.97%)	2.53% (2.30%–2.77%)	5.06% (4.62%–5.41%)	1.97% (1.64%–2.44%)	1.97% (1.8%–2.16%)	5.06% (4.65%–5.37%)	[2, 38]
Fracture	1.09% (1.03%–1.28%)	1.74% (1.59%–1.90%)	2.8% (2.56%–2.99%)	0.96% (0.80%–1.19%)	1.74% (1.59%–1.91%)	5% (4.59%–5.31%)	[2, 20, 38]
Endometrial cancer	0.98% (0.93%–1.15%)	0.98% (0.89%–1.07%)	0.32% (0.3%–0.35%)	1.8% (1.5%–2.23%)	1.8% (1.65%–1.98%)	0.59% (0.55%–0.63%)	[2, 16, 21]
Osteoporosis	13.7% (12.96%–16.10%)^3^	21.9% (19.93%–23.93%)	13.71% (12.52% −14.66%)	12.07% (10.04%–14.92%)	22.9% (20.94%–25.15%)	14.48% (13.31%–15.37%)	[2, 21, 40]
Total	23.18% (21.92%–27.24%)^2^	31.3% (28.48%–34.21%)	24.58% (22.45%–26.28%)	24.68% (20.54%–30.51%)	34.88% (31.90%–38.31%)	27.95% (25.68%–29.66%)	[18, 19, 20, 35, 36]
Probability of grade 1–2 adverse events by regimen and treatment duration^2^, ^3^, ^4^							
Treatment duration	5 years (0 to 5)		10 years (0 to 10)				
Regimen	tamoxifen	tamoxifen +ovarian suppression	aromatase inhibitor	tamoxifen	tamoxifen +ovarian suppression	aromatase inhibitor	
Adverse event							
Musculoskeletal symptoms, fatigue, hot flashes, etc.	71.67% (68.89%–74.45%)	67.06% (64.16%–69.97%)	53.4% (51.64%–55.15%)	86.43% (82.52%–92.9%)	80.88% (77.47% −84.49%)	64.4% (61.3%–67.51%)	[18, 19, 20, 21, 37]
Toxicity grade‐specific utility (range) / duration						Expert opinion	
*Grade 1*							
Dyspareunia, fatigue, hot flashes, etc.	0.9 (0.85–0.95)			1 year (6 months‐full treatment duration)			
*Grade 2*							
Musculoskeletal symptoms, etc.	0.85 (0.75–0.95)			1 year (6 months‐full treatment duration)			
*Grade 3*							
Osteoporosis	0.9 (0.85–0.95)			Treatment duration 5 or 10 years)			
Fractures	0.8 (0.7–0.9)			1 year			
Endometrial cancer	0.85 (0.75–0.95)			1^st^ year of treatment			
Continuing care, endometrial cancer	0.9 (0.85–0.95)			2^nd^ year to lifetime			
*Grade 4*							
Pulmonary embolism	0.65 (0.6–0.7)			9 months (6–12 months)			
New cardiac condition	0.8 (0.7–0.9)			Lifetime			
Stroke	0.7 (0.6–0.8)			1^st^ year of treatment			
Continuing care, stroke	0.85 (0.75–0.95)			2^nd^ year to lifetime			

^1^ We used data about the numbers of breast cancer deaths from online Appendix 7 from the NRG Oncology/NSABP B‐42 trial (Reference [18]) as a source for this input. There were 46 breast cancer deaths in 1983 women receiving AIs after 5 years of prior hormonal therapy (97.68% survival rate) and 47 breast cancer deaths among the 1983 women in the placebo group (97.63%). These rates yield a hazard of breast cancer death of 0.00335 and 0.00343, respectively, for a ratio of 0.978. Assuming an exponential survival function (i.e., constant hazard of breast cancer death over the time period), we derived the reduction in the probability of breast cancer death as HR =0.54. The 95% CI cannot be estimated without additional data not available in the published study.

^2^ The probability of individual adverse events was adjusted proportionally when the range was varied.

^3^ For simplicity, the probability of each adverse event was assumed to be equal across all age groups.

^4^ Of the patients who experienced grade 1–2 adverse events, on average, each patient had two grade 1–2 adverse events.

We selected women diagnosed with invasive ER+, non‐metastatic cancer (AJCC v6 stages 1–3) from this population sample to study the effects of hormonal therapy duration. Systemic treatment for these women was based on age, stage, and molecular‐subtype: (a) women with stage 1 cancer received endocrine therapy (tamoxifen or aromatase inhibitor based on age <50 or 50+, respectively), (b) women <50 with stage 2a, 2b, or 3 cancer received endocrine therapy with tamoxifen and ovarian suppression and all received chemotherapy, and (c) women ages 50+ with stage 2a, 2b, or 3 cancer received endocrine therapy with an aromatase inhibitor plus chemotherapy. All women with HER2+ tumors received trastuzumab. Stages were summarized based on usual CISNET model analyses that approximately map stage to nodal status (stages 1 and 2a were considered node‐negative and stages 2b and 3 as node‐positive based on prognostic mapping for low‐ and high‐risk cancers).

We assumed 100% use of all therapies to estimate efficacy. Treatment efficacy was based on published clinical trials and meta‐analyses and modeled as reductions in the hazard of breast cancer mortality.^2^, ^16^, ^17^ There are not yet sufficient data on the actual rates of the completion of 10 years of endocrine therapy, so alternative assumptions were examined in the sensitivity analysis.

Two types of endocrine therapy‐related side effects were considered. The first had an effect on the quality of life (e.g., musculoskeletal and other grade 1–2 toxicities), but did not affect life expectancy. The second type, such as endometrial cancers or pulmonary embolism, reduced the quality of life (grade 3–4 toxicities) and could have been life‐threatening (grade 5 toxicity). The chance of developing any adverse event was based on a probability distribution derived from reported rates in clinical trials.^18^, ^19^, ^20^, ^21^ The utility weights for these events were based on published studies and expert opinion.^11^, ^22^, ^23^ The impact of adverse events was estimated by applying utility weights over the duration of the adverse event to calculate quality‐adjusted life years (QALYs). Reductions in the quality of life related to endocrine therapy were considered together with utility weights for age‐specific general health and cancer stage^24^ ; reductions in the quality of life related to chemotherapy and HER2‐targeted drugs are captured in the stage‐specific utility. Competing non‐breast cancer mortality was based on age‐ and cohort‐specific data.25

### 2.3 Analysis

We used a lifetime horizon to capture results for women in the upper tail of the life expectancy distribution.^25^ The base case analyses examined lifetime undiscounted and 3% discounted LYs and QALYs, and lifetime probability of breast cancer death by endocrine therapy duration. Discounted QALYs were the primary outcomes; discounting values current more than future LYs. We also calculated age‐ and nodal status‐specific outcomes. We simulated 2 billion women to minimize stochastic variation.

### 2.4 Sensitivity analysis

First, we evaluated a 15‐year time horizon to compare model undiscounted outcomes to selected trial results. Second, we varied parameters related to endocrine therapy adverse events (utility, probability, and duration of adverse events) across the highest to lowest observed values. Third, we tested the effects of varying endocrine therapy completion rates from 100% to 50%, 70%, or 90% by reducing both treatment efficacy and probability of adverse events.^17^ Two‐way sensitivity analyses were conducted to capture: (1) the longest durations of adverse events and lowest utility values (worst case) and (2) the shortest durations of adverse events and highest utility values (best case). A tornado diagram was used to display the results of the sensitivity analyses. Probabilistic sensitivity analysis was not feasible based on computational capability.

### 2.5 Model validation

We verified coding by entering extreme parameter values to ensure the output varied in the expected directions. Next, to assess predictive validity, we used US screening and molecular subtype‐specific treatment dissemination data12 to compare age‐adjusted incidence and mortality rates for women with ER+invasive cancers to SEER data from 2008 to 2017, the last reported date at the time of analyses.^1^ Finally, the sensitivity analysis above comparing short‐term outcomes to clinical trials served as indicators of external validity.

## 3 RESULTS

Projected age‐adjusted breast cancer incidence and mortality rates closely matched observed rates (Supplemental Figure 1, Panels A and B).

**FIGURE 1:**
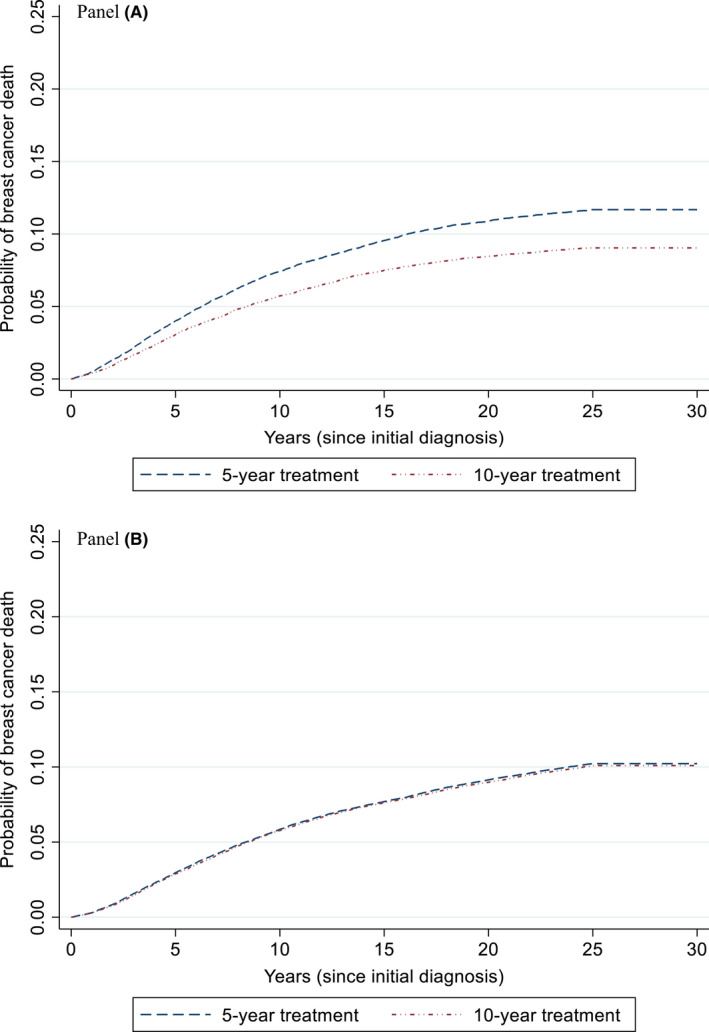
Lifetime probability of breast cancer death among women ages 25–79 with estrogen receptor‐positive, stage 1–3 breast cancers by the duration of adjuvant endocrine therapy. Panel A. Women ages 25–49 treated with tamoxifen (with and without ovarian suppression). Among women 25–49, the difference in the probability of breast cancer mortality for the use of 10 versus 5 years of tamoxifen at 15 and 25‐years post‐diagnosis was 2.13% and 2.63%, respectively. There is no difference in the first 5 years and no additional breast cancer mortality occurred after 25 years. Panel B. Women ages 50–79 treated with aromatase inhibitors. Among women ages 50–79, the difference in the probability of breast cancer mortality with 10 versus 5 years of aromatase inhibitors at 15 and 25 years post‐diagnosis was 0.1% and 0.16%, respectively. There are no mortality differences after 25 years

### 3.1 Effects of extending endocrine therapy

Extending the duration of endocrine therapy with tamoxifen among women ages 25–49 years reduced the lifetime probability of breast cancer death from 11.9% to 9.3% (absolute difference 2.6%; Figure 1, Panel A); the absolute reduction at 15 years was 2.1% (Supplemental Table 1). The lifetime results translate into a gain of 0.77 LYs (281 days)/woman in this age group (undiscounted); adverse events reduced this gain to 0.46 QALYs/woman (undiscounted). Discounted results, which capture the current value of future gains, were similar, but of a smaller magnitude (Table 2).

**TABLE 2:** Lifetime treatment outcomes by endocrine therapy duration by age and regimen among women with estrogen receptor‐positive, non‐metastatic invasive breast cancer

	Tamoxifen among women ages 25 to 49 (with and without ovarian suppression)^1^			Aromatase Inhibitors among women ages 50–79		
Treatment duration	5 years	10 years	Absolute difference	5 years	10 years	Absolute difference
Breast cancer mortality^2^	11.91%	9.28%	2.63% reduction	10.20%	10.04%	0.16% reduction
Undiscounted LYs	35.42	36.19	0.77	20.48	20.48	0.00
Discounted LYs^3^	20.20	20.57	0.37	14.04	14.05	0.01
Undiscounted QALYs	24.21	24.67	0.46	13.36	13.29	−0.07
Discounted QALYs^3^	13.97	14.17	0.20	9.18	9.12	−0.06

Abbreviations: QALYs, quality‐adjusted life years.

^1^ Ovarian suppression was prescribed to the women ages 25–49 with node‐positive cancers.

^2^ Based on the time‐to‐event..

^3^ Discounted at 3%.

Extending endocrine therapy with aromatase inhibitors among women ages 50–79 had a small absolute reduction in the lifetime probability of breast cancer death (0.16%), with a reduction of 0.10 at 15 years (Figure 1, Panel B, and Table 2). Lifetime gains were offset by adverse events (loss of 0.07 QALYs, undiscounted; Table 2).

### 3.2 Effects of extending endocrine therapy by age and nodal status

The results varied across age and nodal status. Women ages 25–49 had a net gain in discounted QALYs with extended tamoxifen therapy after considering adverse effects. However, women ages 50–79 had no net benefit of using aromatase inhibitors in years 5–10 (Table 3). There were greater gains with extended endocrine therapy for all ages of women with node‐positive versus negative cancers, but only women ages 25–49 and 50–59 had a net gain in discounted QALYs (Table 3). Results for LYS followed the same patterns (Supplement Table 2).

**TABLE 3:** Quality‐adjusted life years (QALYs)^1^ by endocrine therapy duration, regimen, age, and nodal status

Treatment duration	5 years	10 years	Absolute difference
Age 25–49 (Tamoxifen with or without ovarian suppression)^2^			
All	13.97	14.17	0.20
Node‐negative	14.58	14.69	0.11
Node‐positive	12.65	13.03	0.39
Age 50–59 (Aromatase inhibitor)			
All	11.41	11.36	−0.05
Node‐negative	11.84	11.78	−0.07
Node‐positive	10.13	10.14	0.01
Age 60–69 (Aromatase inhibitor)			
All	8.91	8.86	−0.05
Node‐negative	9.19	9.11	−0.07
Node‐positive	7.90	7.90	0.00
Age 70–79 (Aromatase inhibitor)			
All	6.50	6.46	−0.04
Node‐negative	6.67	6.63	−0.04
Node‐positive	5.75	5.69	−0.07

^1^ Discounted QALYS; a negative sign indicates a loss in QALYs.

^2^ Ovarian suppression was prescribed to the women ages 25–49 with node‐positive cancers.

### 3.3 Sensitivity analysis

**FIGURE 2:**
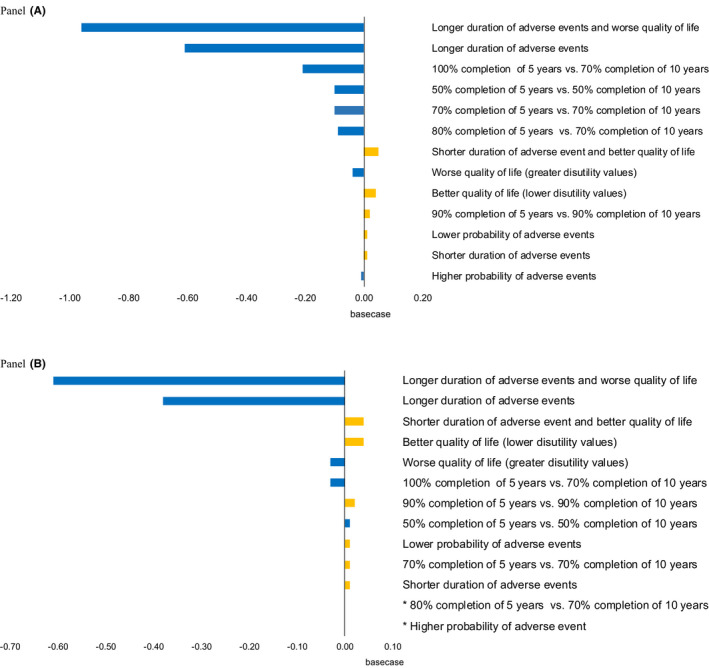
Results of sensitivity analysis testing effects of input parameter uncertainty on results. Panel A. Women ages 25–49 treated with tamoxifen (with and without ovarian suppression). Panel B. Women ages 50–79 treated with aromatase inhibitors. The incremental discounted QALYs between 5 and 10 years of endocrine therapy for each one‐ or two‐way sensitivity analysis. The vertical line indicates the base case incremental discounted QALYs for 5 versus 10 years of therapy. The bars with positive values to the right of the vertical line indicate gains in QALYs compared to the base case. The bars with negative values to the left of the vertical indicate losses in QALYs compared to the base case. ^*^These sensitivity analyses gave the results identical or nearly identical to the base case (see Tables S3 and S4)

The results for all age groups were sensitive to assumptions about the duration of adverse events and utility weights for adverse treatment events (Figure 2, Panels A and B, and Tables S3 and S4). For example, if the duration of adverse events was the longest in the observed range, the QALY gain in the base case was reversed to a QALY loss. The results also varied based on assumptions about the proportion of women that completed therapy. If there were higher completion rates (100% to 80%) in the first 5 years and lower rates (70% to 50%) in the second 5 years, there were fewer net benefits of extended treatment than in the base case.

## 4 DISCUSSION

We used an established simulation model to extend the results of clinical trials to estimate the lifetime efficacy of extended endocrine therapy in groups of women with estrogen receptor‐positive tumors. We found that there were small absolute gains in LYs by extending endocrine therapy from 5 to 10 years among younger women using tamoxifen, but fewer gains for extending aromatase inhibiter use in older women. There were greater benefits with extended endocrine therapy for women with node‐positive versus negative cancers, but only women ages 25–49 and 50–59 had a net gain in QALYs. These conclusions were sensitive to assumptions about the duration and magnitude of adverse treatment‐related effects and rates of actual real‐world treatment completion.

The model results extend current decision tools^26^, ^27^, ^28^ that use genomic and/or clinicopathological data to predict distant recurrence risk by estimating the lifetime effects of extended endocrine therapy, considering adverse effects, age, clinicopathological data, and past screening. Our results also extend clinical trial results since the trials were performed in several different countries and did not consider the quality of life or a lifetime horizon. The overall absolute lifetime benefits of 10 versus 5 years of endocrine therapy projected by the model by age and nodal status were consistent with the summary of current trial evidence used to develop the 2019 ASCO guidelines.^5^

The small benefits seen in the trials and the model can translate into important population‐level mortality reductions. At the individual level, when average benefits are small, it is important to consider sub‐group effects and weigh benefits against known adverse treatment effects. For some women, we found that small lifetime benefits of extended endocrine therapy may be outweighed by the impact of adverse events, especially for older women with short life expectancies due to competing mortality. Older women diagnosed with node‐negative cancers are at comparatively lower risk of distant recurrence than those with node‐positive disease, but the risk of adverse events is similar across nodal groups. Therefore, older women with early‐stage, node‐negative ER+cancers have less net benefit than those with node‐positive disease. Overall, our results underscore the need to consider individual risk and preferences during shared decision‐making discussions about extended therapy, especially for women considering treatment with aromatase inhibitors.

This study used a well‐established simulation model and best modeling practices,^29^ but several limitations should be considered in evaluating the results. First, we estimated treatment efficacy by assuming 100% completion. Rates will be lower in actual community practice,^6^, ^30^, ^31^ and lower treatment completion decreases the absolute benefits of extended endocrine therapy. Second, we did not model second contralateral breast cancers. This underestimates the effects of extending therapy to 10 years, especially since the disease‐free survival benefits of aromatase inhibitors in NSABP B42 were largely due to the avoidance of second primaries.^18^ Third, we did not include the risk of distant recurrence or gene expression profile (GEP) test score results. ^26^, ^27^, ^32^ In particular, tests such as the Breast Cancer Index (BCI) have been used to predict distant recurrence after 5 years of endocrine therapy.^26^ Several studies (e.g., MA17 and IDEAL) suggest that women with high recurrence risk tumors are likely to benefit from extending therapy with aromatase inhibitors.^20^ The fourth limitation is that we did not model changes in regimens from pre‐ to post‐menopause or all possible combinations of regimens due to primary data limitations. In prior trials and clinical practice, there are a number of possible endocrine therapy regimens (e.g., single‐agent tamoxifen or AI vs. sequential combinations), so that the effects of a particular regimen may have been diluted in our model. This may be especially true if the benefits accrue mainly to those with high BCI values. Evolving studies using BCI or other genomic data are likely to more clearly define the specific subsets of women that will benefit from extended endocrine therapy. It will be important to update our model inputs in the future with any new evidence. Fifth, we used age as a proxy for pre‐ and post‐menopausal status. Sixth, nearly 50% of women newly diagnosed with breast cancer in the United States are age 65 and older.^1^ While we used age‐specific competing non‐breast cancer mortality rates, we did not consider the heterogeneity in comorbidities within this age group.^33^ This will be important to include in future modeling. Finally, our analyses were designed to inform clinical decision making so we did not consider costs or include results from multiple models; these will be useful in future analyses examining population outcomes.

Overall, this study illustrates how simulation modeling can be useful to provide a quantitative summary of the net lifetime effects of extended hormonal treatment and adverse effects by age and nodal status.^5^ The lifetime results confirm the ASCO 2019 recommendation that older women with node‐negative low‐risk tumors can likely forego extended aromatase inhibitor therapy. This study also illustrates that the risks and side effects of extended therapy should be weighed against the potential absolute benefits of longer treatment in a shared decision‐making process. Simulation modeling can ultimately be useful as an engine for future clinical decision tools to support oncologists and their patients in making personalized treatment decisions and to highlight subgroups with more benefits than harms by regimen, especially in this era of rapidly expanding knowledge about tumor genomic profiles and recurrence risk.

## ETHICS STATEMENT

The Georgetown University Medical Center Institutional Review Board approved this modeling research as exempt based on the use of de‐identified, publicly available data.

## DISCLOSURES

Young Chandler, Jinani Jayasekera, Allison Kurian, Christopher Cadham, and Jeanne Mandelblatt have no relationships to disclose; Clyde Schechter has an advisory role with COHRDATA and Claudine Isaacs has an advisory role with Pfizer, Novartis, and AstraZeneca.

## CONFLICT OF INTEREST

None.

## AUTHOR CONTRIBUTIONS

Young Chandler, Jeanne Mandelblatt, Jinani Jayasekera, and Clyde Schechter were responsible for the conceptualization, methods, analysis, writing, and editing. Allison Kurian and Claudine Issacs were responsible for clinical concepts and data, writing, and final editing; Christopher Cadham was responsible for data acquisition, analyses, writing, and editing. Jeanne Mandelblatt and Clyde Schechter were responsible for supervision.

## Supporting information

Supplementary Material

## Data Availability

Detailed information about the model is available at https://cisnet.cancer.gov/breast/profiles.html and in the references, but code or executables are not presently publicly available. Output data from the models are available from Dr. Chandler at young.chandler@georgetown.edu. Please contact Dr. Mandelblatt at mandelbj@georgetown.edu to collaborate on using the model for new analyses.
