# Supplementary material for: Simulation modeling of breast cancer endocrine therapy duration by patient and tumor characteristics
Source: Cancer Med. 2021 Dec 16;11(2):297–307. doi: 10.1002/cam4.4084 (PMC8729060; doi:10.1002/cam4.4084)
Supplement: Supplementary file 1 — Supplementary Material [file CAM4-11-297-s001.docx]

**Supplemental Materials**

Supplement Figure 1

Panel A. Model Projected Age-Adjusted* Breast Cancer Incidence Vs. Observed SEER Rates for US Women Age 25-79 with ER-Positive Invasive, Non-Metastatic Breast Cancers

*Age-adjusted to the 2000 US Standard Population

Panel 1B. Model Projected Age-Adjusted* Breast Cancer Mortality Vs. Observed SEER Rates for US Women Age 25-79 with ER-Positive Invasive, Non-Metastatic Breast Cancers

*Age-adjusted to the 2000 US Standard Population

Supplement Table 1. 15-year Outcomes by Endocrine Therapy Duration By Age and Regimen Among Women with Estrogen Receptor Positive, non-Metastatic Breast Cancer

|  | **Tamoxifen**  **among women 25 to 49 (with or without ovarian suppression)^1^** | | | **Aromatase Inhibitors**  **among women age 50-79** | | |
| --- | --- | --- | --- | --- | --- | --- |
| **Treatment duration** | **5-year** | **10-year** | **Absolute difference** | **5-year** | **10-year** | **Absolute difference** |
| Breast cancer mortality ^2^ | 9.79% | 7.66% | 2.13% reduction | 7.69% | 7.59% | 0.1% reduction |
| Undiscounted life years | 13.95 | 14.12 | 0.18 | 12.49 | 12.50 | 0.01 |
| Discounted life years | 11.16 | 11.30 | 0.13 | 10.10 | 10.11 | 0.01 |
| Undiscounted QALYs | 9.84 | 9.91 | 0.06 | 8.24 | 8.18 | -0.06 |
| Discounted QALYs | 7.86 | 7.90 | 0.04 | 6.66 | 6.60 | -0.05 |

QALYs=quality-adjusted life years

1. Ovarian suppression was prescribed to the women age 25-49 with node positive cancers

2. Based on the time to event

Supplemental Table 2A. Discounted Life Years (LYs)^1^ by Endocrine Therapy

Duration, Regimen, Age and Nodal Status

| **Treatment duration** | **5-year** | **10-year** | **Absolute**  **difference** |
| --- | --- | --- | --- |
| **Age 25-49 (Tamoxifen with or without ovarian suppression)**^2^ | | | |
| All | 20.20 | 20.57 | 0.37 |
| Node-negative | 20.73 | 20.97 | 0.24 |
| Node-positive | 19.05 | 19.71 | 0.66 |
| **Age 50-59 (Aromatase inhibitor)** | | | |
| All | 17.03 | 17.06 | 0.02 |
| Node-negative | 17.42 | 17.42 | 0.00 |
| Node-positive | 15.92 | 15.99 | 0.08 |
| **Age 60-69 (Aromatase inhibitor)** | | | |
| All | 13.73 | 13.74 | 0.01 |
| Node-negative | 13.97 | 13.95 | -0.02 |
| Node-positive | 12.85 | 12.95 | 0.10 |
| **Age 70-79 (Aromatase inhibitor)** | | | |
| Stage 1─3 | 10.38 | 10.40 | 0.01 |
| Node-negative | 10.52 | 10.54 | 0.02 |
| Node-positive | 9.77 | 9.74 | -0.03 |

^1^ Discounted LYS; a negative sign indicates a loss in LYS

^2^ Ovarian suppression was prescribed to the women age 25-49 with node positive cancers

Supplemental Table 2B. Undiscounted Life Years (LYs)^1^ by Endocrine Therapy

Duration, Regimen, Age and Nodal Status

| **Treatment duration** | **5-year** | **10-year** | **Absolute**  **difference** |
| --- | --- | --- | --- |
| **Age 25-49 (Tamoxifen with or without ovarian suppression)**^2^ | | | |
| All | 35.42 | 36.19 | 0.77 |
| Node-negative | 36.43 | 36.92 | 0.49 |
| Node-positive | 33.24 | 34.62 | 1.37 |
| **Age 50-59 (Aromatase inhibitor)** | | | |
| All | 26.59 | 26.62 | 0.03 |
| Node-negative | 27.25 | 27.25 | 0.00 |
| Node-positive | 24.64 | 24.78 | 0.13 |
| **Age 60-69 (Aromatase inhibitor)** | | | |
| All | 19.51 | 19.52 | 0.01 |
| Node-negative | 19.88 | 19.85 | -0.03 |
| Node-positive | 18.12 | 18.28 | 0.16 |
| **Age 70-79 (Aromatase inhibitor)** | | | |
| All | 13.52 | 13.54 | 0.02 |
| Node-negative | 13.72 | 13.75 | 0.03 |
| Node-positive | 12.65 | 12.60 | -0.05 |

^1^  A negative sign indicates a loss in LYS

^2^ Ovarian suppression was prescribed to the women age 25-49 with node positive cancers

Supplement Table 3. Sensitivity Analysis Results for Women Ages 25-49 with Tamoxifen Therapy (with or Without Ovarian Suppression)

| **Range of Input Parameters** | **5-year QALYs** | **10-year**  **QALYs** | **Absolute difference^1^** |
| --- | --- | --- | --- |
| ***Utility Weight Values*** | | | |
| Better quality of life (less dis-utility values) | 14.13 | 14.37 | 0.24 |
| Worse quality of life (more dis-utility values) | 13.81 | 13.97 | 0.16 |
| ***Probability of Adverse Event*** | | | |
| Higher probability of adverse event | 13.94 | 14.13 | 0.19 |
| Lower probability of adverse event | 13.99 | 14.20 | 0.21 |
| ***Treatment Completion*** | | | |
| 90% completion of 5 years vs. 90% completion of 10 years^2^ | 13.92 | 14.13 | 0.22 |
| 70% completion of 5 years vs. 70% completion of 10 years^2^ | 13.81 | 13.92 | 0.10 |
| 50% completion of 5 years vs. 50% completion of 10 years^2^ | 13.81 | 13.92 | 0.10 |
| 100% completion of 5 years vs. 70% completion of 10 years^3^ | 14.00 | 14.00 | -0.01 |
| 80% completion of 5 years vs. 70% completion of 10 years^4^ | 13.88 | 14.00 | 0.11 |
| ***Duration of Adverse Event*** | | | |
| Longer duration of adverse event(s) ^5^ | 13.56 | 13.16 | -0.41 |
| Shorter duration of adverse event(s) ^6^ | 14.02 | 14.23 | 0.21 |
| ***Combination of Various Durations of Adverse Event with Better/Worse Quality of Life*** | | | |
| Longer duration of adverse event and worse quality of life | 13.20 | 12.44 | -0.76 |
| Shorter duration of adverse event and better quality of life | 14.15 | 14.40 | 0.25 |

1. A negative sign indicates a loss in QALYs
2. The same suboptimal treatment completion for both 5 and 10 years of endocrine therapy, under the assumption of proportional reduction in treatment effect and probability of adverse event(s)
3. 100% treatment completion for 5 years of endocrine therapy and 70% of treatment completion for 10 years, under the assumption of proportional reduction in treatment effect and probability of adverse event(s)
4. 80% treatment completion for 5 years of endocrine therapy and 70% of treatment completion for 10 years, under the assumption of proportional reduction in treatment effect and probability of adverse event(s)
5. The duration of grade 1-2 adverse event was 5 or 10 years (same with the treatment duration). The duration of pulmonary embolism was 12 months.
6. The duration of grade 1-2 adverse event was 6 months. The duration of pulmonary embolism was 6 months.

Supplement Table 4. Sensitivity Analysis Results for Women Ages 50-79 with Aromatase Inhibitor Therapy

| **Range of Input Parameters** | **5-year QALYs** | **10-year**  **QALYs** | **Absolute difference^1^** |
| --- | --- | --- | --- |
| ***Utility Weight Values*** | | | |
| Better quality of life (less dis-utility values) | 9.31 | 9.29 | -0.02 |
| Worse quality of life (more dis-utility values) | 9.05 | 8.96 | -0.09 |
| ***Probability of Adverse Event*** | | | |
| Higher probability of adverse event | 9.16 | 9.10 | -0.06 |
| Lower probability of adverse event | 9.20 | 9.14 | -0.05 |
| ***Treatment Completion*** | | | |
| 90% completion of 5 years vs. 90% completion of 10 years^2^ | 9.16 | 9.12 | -0.04 |
| 70% completion of 5 years vs. 70% completion of 10 years^2^ | 9.13 | 9.08 | -0.05 |
| 50% completion of 5 years vs. 50% completion of 10 years^2^ | 9.11 | 9.06 | -0.05 |
| 100% completion of 5 years vs. 70% completion of 10 years^3^ | 9.17 | 9.08 | -0.09 |
| 80% completion of 5 years vs. 70% completion of 10 years^4^ | 9.14 | 9.08 | -0.06 |
| ***Duration of Adverse Event*** | | | |
| Longer duration of adverse event(s) ^5^ | 8.90 | 8.46 | -0.44 |
| Shorter duration of adverse event(s) ^6^ | 9.22 | 9.17 | -0.05 |
| ***Combination of Various Durations of Adverse Event with Better/Worse Quality of Life*** | | | |
| Longer duration of adverse event and worse quality of life | 8.63 | 7.96 | -0.67 |
| Shorter duration of adverse event and better quality of life | 9.33 | 9.31 | -0.02 |

1. A negative sign indicates a loss in QALYs
2. The same suboptimal treatment completion for both 5 and 10 years of endocrine therapy, under the assumption of proportional reduction in treatment effect and probability of adverse event(s)
3. 100% treatment completion for 5 years of endocrine therapy and 70% of treatment completion for 10 years, under the assumption of proportional reduction in treatment effect and probability of adverse event(s)
4. 80% treatment completion for 5 years of endocrine therapy and 70% of treatment completion for 10 years, under the assumption of proportional reduction in treatment effect and probability of adverse event(s)
5. The duration of grade 1-2 adverse event was 5 or 10 years (same with the treatment duration). The duration of pulmonary embolism was 12 months.
6. The duration of grade 1-2 adverse event was 6 months. The duration of pulmonary embolism was 6 months.
